# Evaluation of the food composition tables: Beyond the divergence of intakes

**DOI:** 10.1111/mcn.13149

**Published:** 2021-03-22

**Authors:** Sabuktagin Rahman, Avonti Basak Tukun, Santhia Ireen, Nazma Shaheen

**Affiliations:** ^1^ Public Health, School of Medicine Griffith University Gold Coast Queensland Australia; ^2^ Institute of Nutrition and Food Sciences University of Dhaka Dhaka Bangladesh; ^3^ Alive and Thrive Initiative fhi360 Bangladesh Program Gulshan 2, Dhaka Bangladesh

Dear Editor,

We ponder upon the study of Ali M. et al (2020) published recently comparing the two local Food Composition Tables (FCTs) of Bangladesh through measuring nutrient intakes of their study. Just assessing the intake differences fall short of the proper evaluation of the FCTs. The evaluation of the FCTs includes its stringent selection, which primarily underscores the data quality rather than the nutrient intake analysis. It is evident that the quality of FCT hinges on methodologies of its development, for example, food species, sample selection, analytical methods used for nutrient estimation, units of reporting and ways for dealing with missing nutrients (Greenfield & Southgate, 2003; Merchant & Dehghan, 2006). Apart from the evaluation issues, we have reservations on the methodologies to study the agreement and conclusion.

The study has some methodological insufficiencies and there was room for improvement assessing the agreement. The de‐attenuated correlation usually improves the energy‐adjusted agreement between the competing dietary methods (Chen et al., 2004; Lin et al., 2017). Bland–Altman Plot analysis—the most commonly used agreement test (Cade et al., 2002) could have shown additional insights into the agreement. Weighted Kappa estimate of <0.2 is considered “poor” (Altman, 1991). The authors reported that only 4 (folate, B6, sodium and magnesium) of the 15 micronutrients fell below the cut‐off. Except for folate none of them is considered of prime importance in Bangladesh public health context. Hence, a sweeping conclusion that the micronutrients values need cautious interpretation using these FCTs seems exaggerated. The local FCTs, such as the FCTB‐2013 has followed the standard procedures and guidelines of FAO/INFOODS (FAO/INFOODS, 2012a; FAO/INFOODS, 2012b; FAO/INFOODS, 2012c) to produce an internationally comparable FCT including primary analysis of 20 key foods (Haytowitz, Pehrsson, & Holden, 2002); and composite samples were collected from 28 out of 30 agro‐ecological zones (Shaheen et al., 2014).

## CONFLICTS OF INTEREST

Authors declare no competing interests.

## CONTRIBUTIONS

SR and NS conceived the idea, SR wrote the first draft; NS, ABT and SI provided feedback and evaluated to finalise. All the authors read and approved the final version.
